# HEALTH EQUITY APPROACH TO EXAMINE COGNITIVE DIFFICULTY WITH AND WITHOUT A MENA CHECKBOX IN THE US

**DOI:** 10.1093/geroni/igae098.3201

**Published:** 2024-12-31

**Authors:** Tiffany Kindratt, Basma Tnesh

**Affiliations:** The University of Texas Arlington, Arlington, Texas, United States; The University of Texas Arlington, Arlington, Texas, United States

## Abstract

Federal guidelines for collecting race/ethnicity in the US include White, Black, Hispanic/Latino, Asian, American Indian/Alaskan Native (AI/AN), and Native Hawaiian/Other Pacific Islander (NH/OPI) as minimum reporting categories. Middle Eastern and North African (MENA) Americans are included in the definition for the White race group, which includes other individuals of European ancestry. In 2023, the Office of Management and Budget proposed adding a separate checkbox for MENA individuals. Several studies have identified that MENA Americans have a higher burden of cognitive health concerns compared to Whites. Having a separate checkbox for MENA Americans may provide a truer assessment of health disparities for other racial/ethnic groups. We examined whether removing MENA Americans from the White race group altered comparisons of cognitive difficulty between racial/ethnic minoritized populations and Whites. Using national data from the American Community Survey (ages >=45 years; N=7,284,988), we identified 67,213 adults (ages 45+ years) with MENA ancestry/birthplace. Most self-reported as White (n=60,993) followed by some other race (n=3,699), Black (n=1,957), Asian (n=468), AI/AN (n=72), and NH/OPI (n=24). Cognitive difficulty was higher among Black, Asian, AI/AN, and NH/OPI compared to White adults before and after removing adults with a MENA ancestry or birthplace from the sample. Marginal changes were observed. For example, for Black adults, odds increased from 1.23 to 1.24. While we did not find a difference in cognitive difficulty when we removed MENA adults from the national sample, further research is needed to determine whether this finding differs in smaller samples (e.g., states) and for other health outcomes.

